# Associations between placental DNA methylation and emotional and behavioral outcomes in preschoolers: insights from the EDEN Mother-Child Cohort study

**DOI:** 10.1192/j.eurpsy.2023.1127

**Published:** 2023-07-19

**Authors:** A. Nakamura, L. Broseus, J. Tost, D. Vaiman, S. Martins, K. Keyes, K. Bonello, M. Fekom, K. Strandberg-Larsen, A.-L. Sutter-Dallay, M. Melchior, B. Heude, J. Lepeule

**Affiliations:** 1 Institute for Advanced Biosciences (IAB), INSERM, La Tronche; 2 Institut de Biologie François Jacob, CEA, Evry; 3 Genomics, Epigenetics and Physiopathology of Reproduction, INSERM, Paris, France; 4 Department of Epidemiology, Columbia University Mailman School of Public Health, New York, United States; 5 Institut Pierre Louis d’Epidémiologie et de Santé Publique (IPLESP), INSERM; 6 School of Medicine, Department of general practice, Sorbonne Université, Paris, France; 7 Section of Epidemiology, Department of Public Health, University of Copenhagen, Copenhagen, Denmark; 8 Bordeaux Population Health, INSERM; 9 University Department of Adult Psychiatry, Charles-Perrens Hospital, Bordeaux; 10 Institut Convergences Migration, CNRS, Aubervilliers; 11 Centre for Research in Epidemiology and Statistics (CRESS), INSERM, Villejuif, France

## Abstract

**Introduction:**

Behavioral (externalizing) and emotional (internalizing) problems were showed to be associated with the prenatal environment. Changes in placental DNA methylation was identified as a relevant potential mechanism of such association.

**Objectives:**

We aimed to explore the associations between placental DNA methylation and child behavior in order to explore pathways that could link prenatal exposures to child behavior.

**Methods:**

Data including 441 children of 3 years of age from the EDEN mother-child cohort. Child behavior assessed using the Strengths and Difficulties Questionnaire (SDQ). Both hypotheses-driven and exploratory analyses (including epigenome-wide association studies (EWAS) and differentially methylated regions (DMR) analyses) were conducted. The analyses were adjusted for confounding and technical factors and estimated placental cell composition. All the p-values were corrected using a false discovery rate (FDR) procedure for multiple tests.

**Results:**

In the hypothesis-driven analysis, *cg26703534* (*AHRR*), was significantly associated with emotional problems (p_FDR_ = 0.03). In the exploratory analyses, *cg09126090* (p_FDR_ = 0.04) and *cg10305789* (*PPP1R16B;* p_FDR_ < 0.01) were significantly associated with peer-relationship problems and 33 DMRs were significantly associated with at least one of the SDQ subscales. Placental DNA methylation showed more associations with internalizing than externalizing symptoms, especially among girls. DMRs tented to include highly methylated CpGs.

**Conclusions:**

This study investigated for the first time the associations between placental DNA methylation and internalizing and externalizing symptoms in preschoolers. Further analyses, such as consortium meta-analyses would be necessary to confirm and extend our results.

**Disclosure of Interest:**

None Declared

